# MiR-27a promotes insulin resistance and mediates glucose metabolism by targeting PPAR-γ-mediated PI3K/AKT signaling

**DOI:** 10.18632/aging.102263

**Published:** 2019-09-28

**Authors:** Tianbao Chen, Yi Zhang, Yilan Liu, Dexiao Zhu, Jing Yu, Guoqian Li, Zhichun Sun, Wanru Wang, Hongwei Jiang, Zhenzhen Hong

**Affiliations:** 1 Department of Cardiology, The First Affiliated Hospital of Quanzhou, Fujian Medical University, Quanzhou, Fujian, China; 2 Department of Endocrinology, The First Affiliated Hospital of Quanzhou, Fujian Medical University, Quanzhou, Fujian, China; 3 Department of Endocrinology, The First Affiliated Hospital of Henan University of Science and Technology, Luoyang, Henan, China

**Keywords:** miR-27a, insulin resistance, glucose metabolism, PPAR-γ, PI3K/AKT

## Abstract

This study aimed to establish a high-fat diet (HFD)-fed obese mouse model and a cell culture model of insulin resistance (IR) in mature 3T3-L1 adipocytes. A dual-luciferase reporter assay (DLRA) was confirmed interaction between miR-27a and the 3′-untranslated region (UTR) of Peroxisome proliferator-activated receptor (PPAR)-γ. The inhibition of PPAR-γ expression by microRNA (miR)-27a in IR cells at both the protein and mRNA levels was confirmed by a mechanistic investigation. Moreover, the 3′-UTR of PPAR-γ was found to be a direct target of miR-27a, based on the DLRA. Furthermore, antagomiR-27a upregulated the activation of PI3K/Akt signaling and glucose transporter type 4 (GLUT4) expression at the protein and mRNA levels. Additionally, the PPAR inhibitor T0070907 repressed the insulin sensitivity upregulated by antagomiR-27a, which was accompanied by the inhibition of PPAR-γ expression and increased levels of AKT phosphorylation and GLUT4. The PI3K inhibitor wortmannin reduced miR-27a-induced increases in AKT phosphorylation, glucose uptake, and GLUT4. miR-27a is considered to be involved in the PPAR-γ-PI3K/AKT-GLUT4 signaling axis, thus leading to increased glucose uptake and decreased IR in HFD-fed mice and 3T3-L1 adipocytes. Therefore, miR-27a is a novel target for the treatment of IR in obesity and diabetes.

## INTRODUCTION

Type 2 diabetes mellitus (T2DM) is a major worldwide health problem affecting not only adults but also children [1, 2]. Over the past 30 years, the occurrence of T2DM has increased constantly, suggesting that an in-depth understanding of its complex pathologic mechanism is urgently needed. The major widely accepted pathogenic factors of T2DM are genetic factors, obesity, lifestyle factors, and impaired glucose tolerance [3, 4]. Impaired glucose metabolism is commonly involved in insulin resistance (IR), which is a critical characteristic of metabolic syndrome [5]. IR is an attenuated capability of targeted cells, such as skeletal muscle cells, hepatocytes, and adipocytes, to respond to insulin stimulation [6]. IR contributes to impaired glycogen synthesis and the failure to repress glucose production in the liver. However, the current understanding of the underlying molecular mechanisms for hepatic IR is still lacking.

MicroRNAs (miRs), a group of non-coding RNA molecules of 22-25 nucleotides in length, can regulate the expression of target genes after transcription, which ultimately leads to the degradation of target mRNA [7]. Accumulating evidence has demonstrated that miRNAs play vital roles in numerous processes at the molecular and biological levels, such as cell proliferation, migration, necrocytosis, and death [8, 9]. In recent years, miRNAs have been proposed to be agents involved in IR caused by hepatitis C, breast cancer, obesity, and T2DM [10–13] because they regulate the activity of several critical signaling pathways, suggesting that miRNAs may be promising targets for the treatment of IR and glucose metabolism [12, 14]. The miRNA miR-27a is one of the most important miRNAs identified to date, owing to its role in the regulation of multiple biological and pathogenic processes, such as pancreatic cancer, gastric cancer, human hepatocellular cancer, and osteoarthritis [15–18], which is a consequence of its ability to target multiple oncogenes. Moreover, in various types of cancer, a reduction of miR-27a expression is associated with a poor prognosis [19]. For insulin sensitivity, miR-27a has been demonstrated to participate in the signaling pathways relevant to glucose metabolism in IR. The altered expression of miR-27a in L6 cells decreases glucose consumption and glucose uptake, and reduces the expression of glucose transporter type 4 (GLUT4), mitogen-activated protein kinase (MAPK)14, and PI3K regulatory subunit beta [20]. The expression of miR-27a in adipose tissue upregulates macrophage activation by inhibiting peroxisome proliferator-activated receptor (PPAR)-γ expression in IR induced by high-fat diet (HFD)-associated obesity [21]. Meanwhile, it has also been reported that adipocyte-derived exosomal miR-27a induces IR in skeletal muscle by repressing PPAR-γ expression [22]. However, its role and molecular mechanism in HFD-fed mice and IR cells have not been fully elucidated.

As an anti-inflammatory factor [23], PPAR-γ can be used to facilitate fatty acid metabolism and reduce the levels of circulating lipids [24]. PPAR-γ activation reduces hyperglycemia by increasing sensitivity to peripheral insulin and decreasing the production of hepatic glucose [25]. Additionally, PPAR-γ plays vital roles in the differentiation and maturation of 3T3-L1 preadipocytes and other fat cells [26]. PPAR-γ is reported to suppress transforming growth factor (TGF)-β signaling, which affects two pro-survival pathways [27]. TGF-β can activate PI3K/Akt signaling by activating p38 MAPK and focal adhesion kinase sensors, which has been demonstrated to regulate IR and sensitivity [28]. In the present study, we established an HFD-fed obese mouse model and a tumor necrosis factor (TNF)-α-induced IR adipocyte cell model to elucidate the influences of miR-27a on IR and glucose metabolism. Our results showed that PPAR-γ served as a direct target for miR-27a in controlling IR and glucose uptake. Therefore, this study suggests that miR-27a is a promising candidate for the treatment of IR in obesity and T2DM.

## RESULTS

### Upregulation of miR-27a levels in an obese mouse model and IR cells

**Figure 1:**
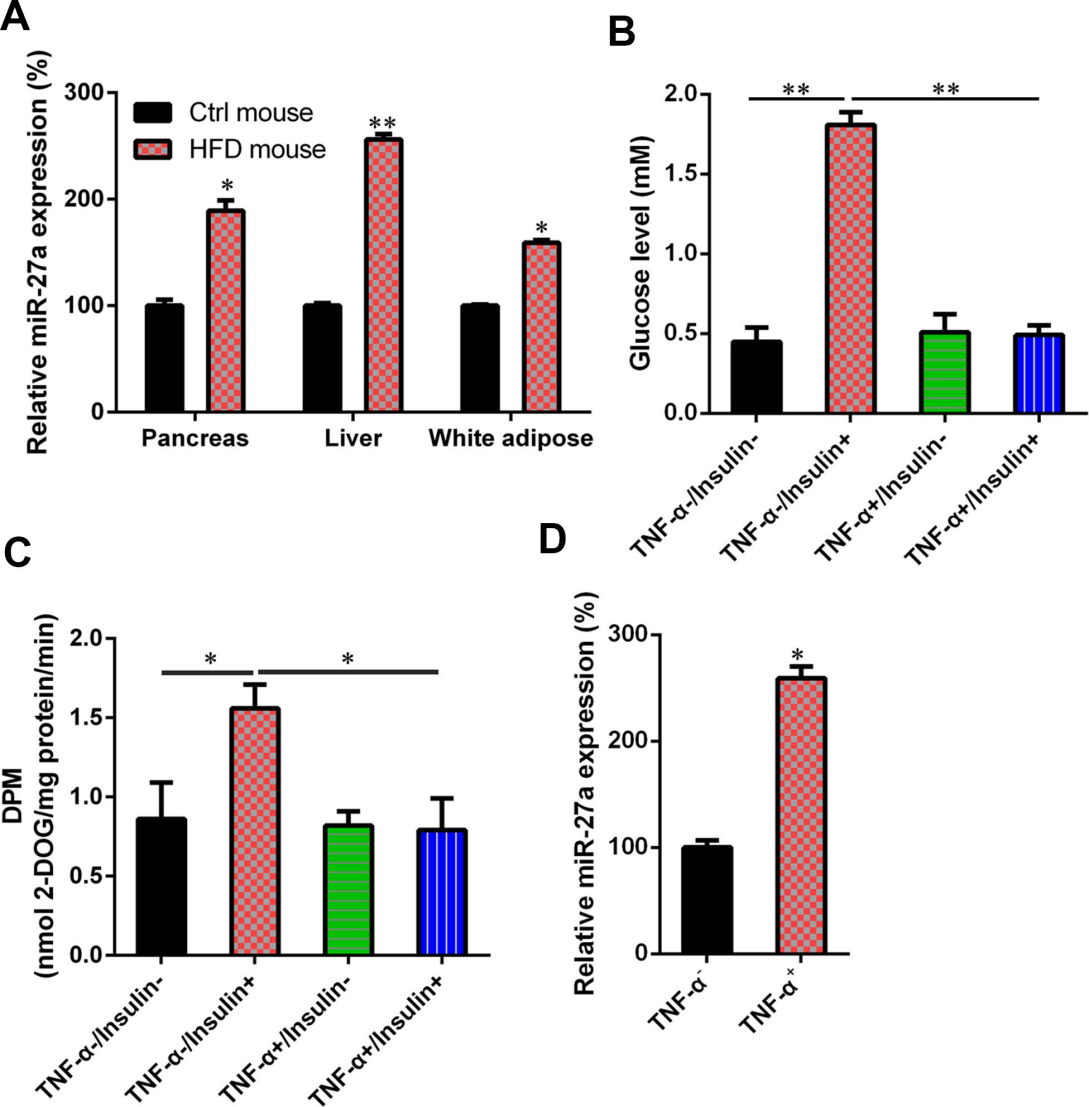
**Upregulation of MiR-27a in HFD-fed mice and IR cells**. (**A**) For obese mice fed with HFD, an increase in miR-27a expression levels in pancreas, liver, and white adipose tissue was revealed using real-time PCR at week 11 after modeling. (**B**) Cells were treated with high-glucose DMEM containing FBS (10%, w/v) supplemented with TNF-α (10 ng/ml) for one day. Subsequently, they were incubated for 0.5 h with another cell medium, i.e., insulin (100 nM) in high-glucose DMEM containing FBS (10%, w/v). The establishment of the IR adipocyte model was confirmed by glucose level results. (**C**) 2-deoxyglucose uptake assay was also performed to detect the glucose level in both cell and cell culture medium. (**D**) qPCR was utilized to showed that the miR-27a levels were obviously increased in the IR cell model treated with TNF-α, compared with those of the normal 3T3-L1 cells. Expression data from each mouse was normalized to that of randomly assigned mouse in control group. Number of animal per group = 8. **P < 0.01, *P < 0.05, compared to indicated groups.

An HFD-induced obese mouse model and IR cell model were established to study the role of miR-27a expression in glucose metabolism and IR. In the obese mice, miR-27a expression in the pancreas, liver, and white adipose tissue was significantly upregulated, compared with normal control mice (Figure 1A). The generation of IR cells, obtained by pre-incubation of 3T3-L1 adipocytes with TNF-α, was confirmed after detecting glucose levels before and after the addition of insulin. As shown in Figure 1B and 1C, in the absence of TNF-α, glucose uptake was increased in the cells after adding insulin. However, no change was observed in glucose uptake after pre-incubation in TNF-α, in either the presence or absence of insulin. This suggested the successful establishment of an IR model in adipocytes. Data from qRT-PCR showed that miR-27a expression was also increased after pre-incubation in TNF-α (Figure 1D).

### Mediation of glucose metabolism and IR in HFD-fed mice and IR 3T3-L1 cells by miR-27a expression

**Figure 2:**
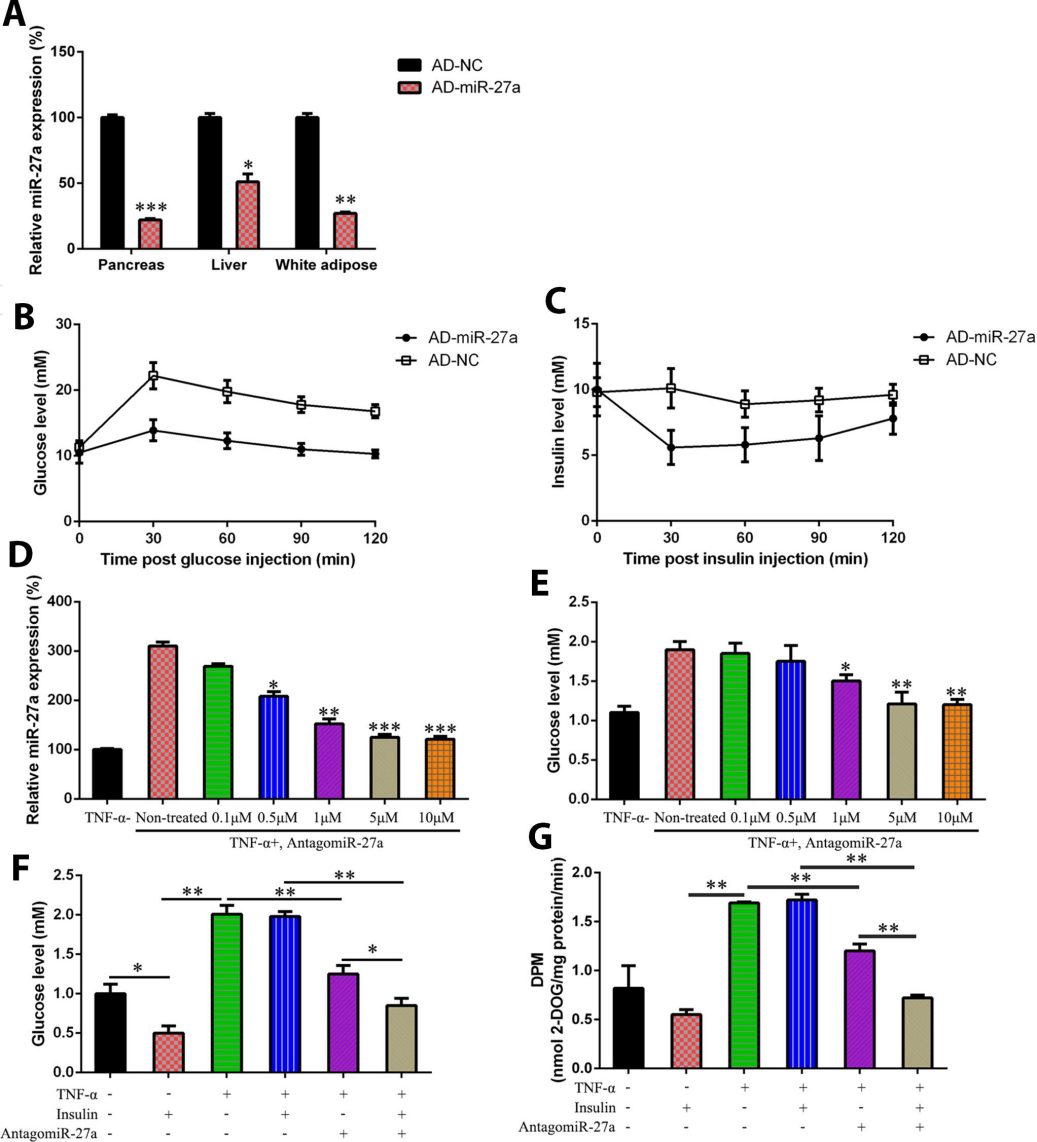
**MiR-27a-induced hyperglycemia amelioration and IR prevention in obese mouse model and IR cells.** C57BL/6 mice were induced with HFD (Research Diet, USA; 45% kcal from fat), or with standard chow diet, both for 10 weeks. The mice were injected intravenously with AD-NC and Ad-miR-27a. (**A**) miR-27a expression levels in pancreas, liver, and white adipose tissue was determined using real-time PCR (**B**) GTT was performed in HFD-fed mice at day 7 after AD-NC or AD-miR-27a injection. (**C**) ITT was performed in HFD-fed mice at 7 days after AD-NC or AD-miR-27a injection. (**D**) In the IR 3T3-L1 cells transfected with antagomiR-27a (different doses), the miR-27a expression level was studied by qPCR. (**E**) Glucose levels were tested in IR 3T3-L1 cells transfected with antagomiR-27a (different doses). (**F**) Glucose levels were tested in IR 3T3-L1 cells transfected with antagomiR-27a (different doses) and/or supplemented with insulin. (**G**) 2-deoxyglucose uptake assay was also performed to detect the glucose level in both cell and cell culture medium. Number of animal per group = 8. ***P < 0.001, **P < 0.01, *P < 0.05, compared to indicated groups.

The HFD-fed mice were injected intravenously via the tail vein with a recombinant adenovirus expressing miR-27a inhibitor (AD-miR-27a) to study the function of miR-27a in glucose metabolism and IR *in vivo*. At first, miR-27a expression was found to be reduced in the pancreas, liver, and white adipose tissue of HFD-fed mice after AD-miR-27a injection compared with the NC group (Figure 2A). Compared with the mice treated with AD-NC, the HFD-fed mice treated with the AD-miR-27a showed a significant reduction in fasting blood glucose levels. Glucose tolerance was also remarkably increased by the reduction of miR-27a expression in a dose-dependent manner, as revealed by an oral glucose tolerance test (Figure 2B). Meanwhile, AD-miR-27a injection improved the insulin sensitivity of the obese mice, as confirmed by their steeper rate of reduced blood glucose levels in response to insulin (Figure 2C).

IR 3T3-L1 cells were transfected with a miR-27a-expressing plasmid at different concentrations. The expression level of miR-27a in each group was determined by qRT-PCR (Figure 2D). The glucose levels in IR 3T3-L1 cells remarkably decreased in the presence of the miR-27a-expressing vector (5 and 10 μM) compared with the non-transfected group (Figure 2E). Moreover, the effects of miR-27a on IR were studied by glucose detection and a 2-DG uptake assay (Figure 2F and 2G). Glucose consumption was increased in the IR cells by miR-27a downregulation after adding insulin, compared with the insulin treatment alone groups. Therefore, miR-27a played a regulatory role in increasing insulin sensitivity.

### MiR-27a targets PPAR-γ

**Figure 3:**
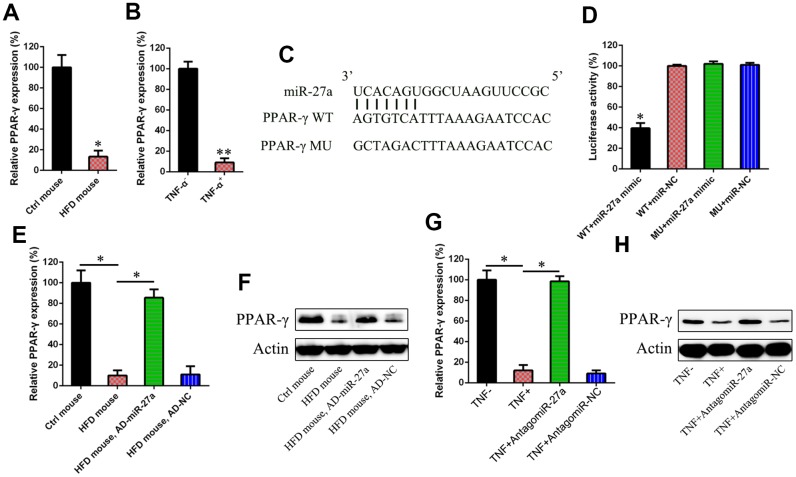
**miR-27a targeted at the 3′-UTR of PPAR-γ.** In the obese mouse model (**A**) and IR cells (**B**), the PPAR-γ expression levels were studied using qPCR. (**C**) A binding site of miR-27a was found in the 3′-UTR of PPAR-γ mRNA, as evidenced by performing bio-informatic analysis results. (**D**) After the co-transfection, one from PPAR-γ to a luciferase reporter containing a wild type (WT) or mutant (MU) 3′-UTR, and the other from agomiR-27a into HEK293T cells, a dual-luciferase reporter assay was performed. Effects agomiR-27a transfection on the luciferase activities of the WT (**E**) and MU (**F**) PPAR-γ reporter constructs were determined. At both the protein and mRNA levels, a sharp increase was observed for the levels of PPAR-γ in the pancreas of HFD-fed mice after injection with AD-miR-27a, when compared with those of the control animals. WB (**G**) and qPCR assay (**H**) showed that miR-27a downregulation obviously increased the expression of both PPAR-γ protein and mRNA. Number of animal per group = 8. **P < 0.01, *P < 0.05, compared to indicated groups.

PPAR-γ expression was studied in the obese mice and IR cells by qRT-PCR, because activated PPAR-γ has been reported to reduce hyperglycemia by increasing peripheral insulin sensitivity and alleviating the production of hepatic glucose [25]. We found that the obese mice and IR cells exhibited reduced PPAR-γ expression (Figure 3A and 3B). Bioinformatics analysis showed that miR-27a may target PPAR-γ (Figure 3C). The direct interaction of miR-27a with WT and MU PPAR-γ was studied using a dual-luciferase reporter assay (DLRA) in which the 3′-UTR of PPAR-γ was fused with a gene encoding luciferase. The results showed that luciferase levels were reduced by 50% after transfection of agomiR-27aand WT 3′-UTR of PPAR-γ compared with the MU groups (Figure 3D). The effects of miR-27a on PPAR-γ expression in HFD-fed mice injected with AD-NC or AD-miR-27a were investigated by WB and qRT-PCR analyses. The protein and mRNA levels of PPAR-γ were obviously increased in the absence of miR-27a (Figure 3E and 3F). IR 3T3-L1 adipocytes were transfected with antagomiR-27a or antagomiR-NC and PPAR-γ production was investigated. The results showed that PPAR-γ expression was augmented after antagomiR-27a transfection (Figure 3G and 3H), suggesting that miR-27a targeted the 3′-UTR of PPAR-γ.

### MiR-27a expression is associated with GLUT4 expression and PI3K/Akt axis activation in IR cells

**Figure 4:**
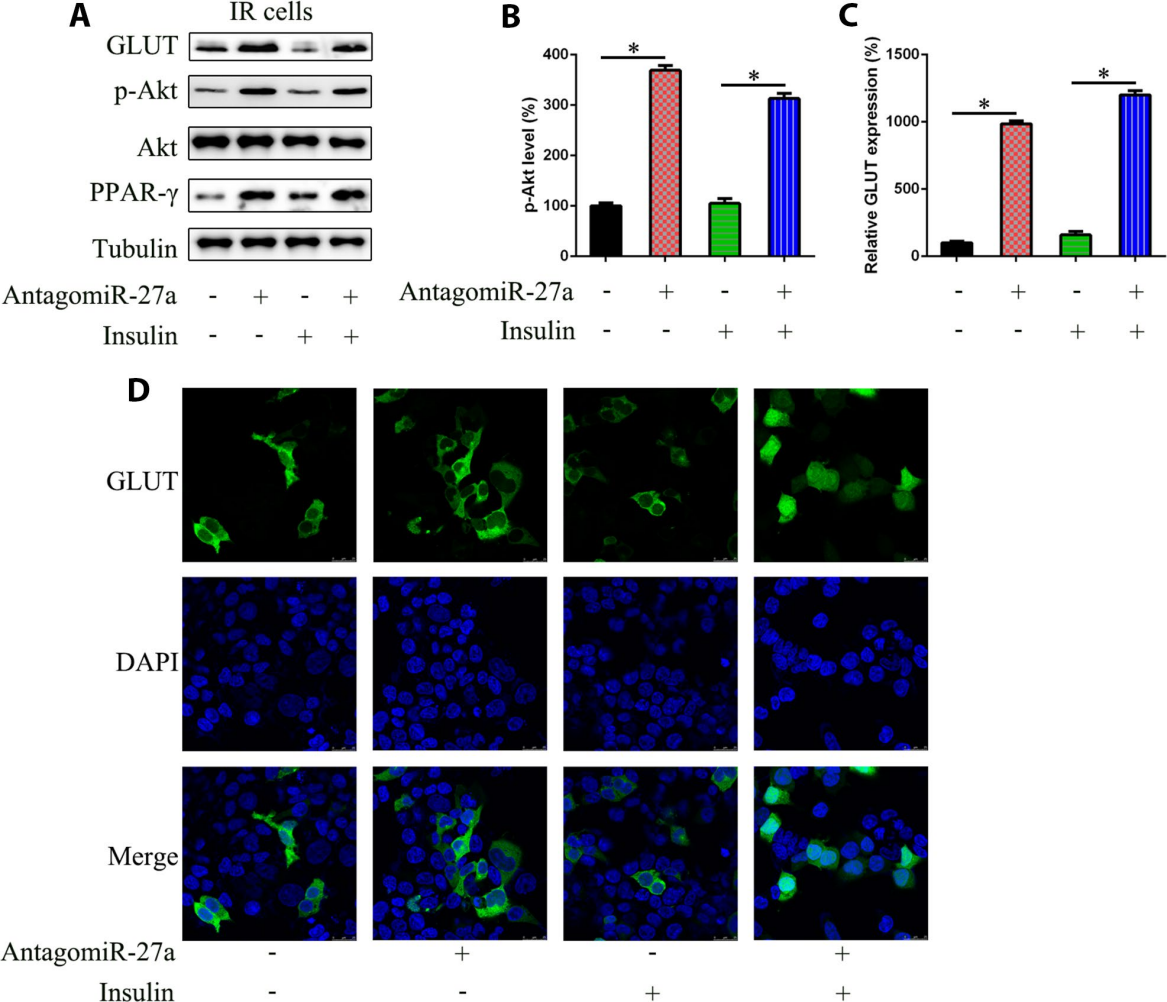
**Effects of miR-27a on the Akt activation and GLUT expression in IR 3T3-L1 cells.** Cells were transfected with antagomiR-27a or antagomiR-NC, and then treated with TNF-α (10 ng/ml) and/or insulin (100 nM) in high-glucose DMEM containing FBS (10%, w/v). (**A**) WB displayed that antagomiR-27a transfection led to GLUT upregulation and PPAR-γ expression and increased Akt phosphorylation, while showing no effect on Akt levels in IR 3T3-L1 cells. (**B**) Image pixel analysis of phosphorylated Akt band. (**C**) qPCR assay showed GLUT mRNA expression in IR 3T3-L1 cells transfected with antagomiR-27a. (**D**) IFA showed that GLUT4 staining was increased after antagomiR-27a transfection. Number = 3. *P < 0.05, compared to indicated groups.

As PPAR-γ has been reported to be an activator of the PI3K/Akt signaling pathway and a regulator of GLUT4 expression, we determined PI3K/Akt signaling activation and GLUT4 expression in IR cells after transfection with antagomiR-27a. Upregulation of PPAR-γ expression was confirmed by WB analysis (Figure 4A), and we observed increased levels of phosphorylated Akt both with and without insulin treatment, suggesting that the PI3K/Akt signaling pathway was activated by miR-27a downregulation (Figure 4B). WB, qRT-PCR, and immunofluorescence assays showed that GLUT4 levels were also increased in antagomiR-27a transfected cells (Figure 4C and 4D). Meanwhile, administration of antagomiR-27a caused an obvious nuclear localization of GLUT4, suggesting that miR-27a is negatively associated with GLUT4 expression via the PPAR-γ-PI3K-Akt axis.

### MiR-27a mediates insulin sensitivity through PPAR-γ activation

**Figure 5:**
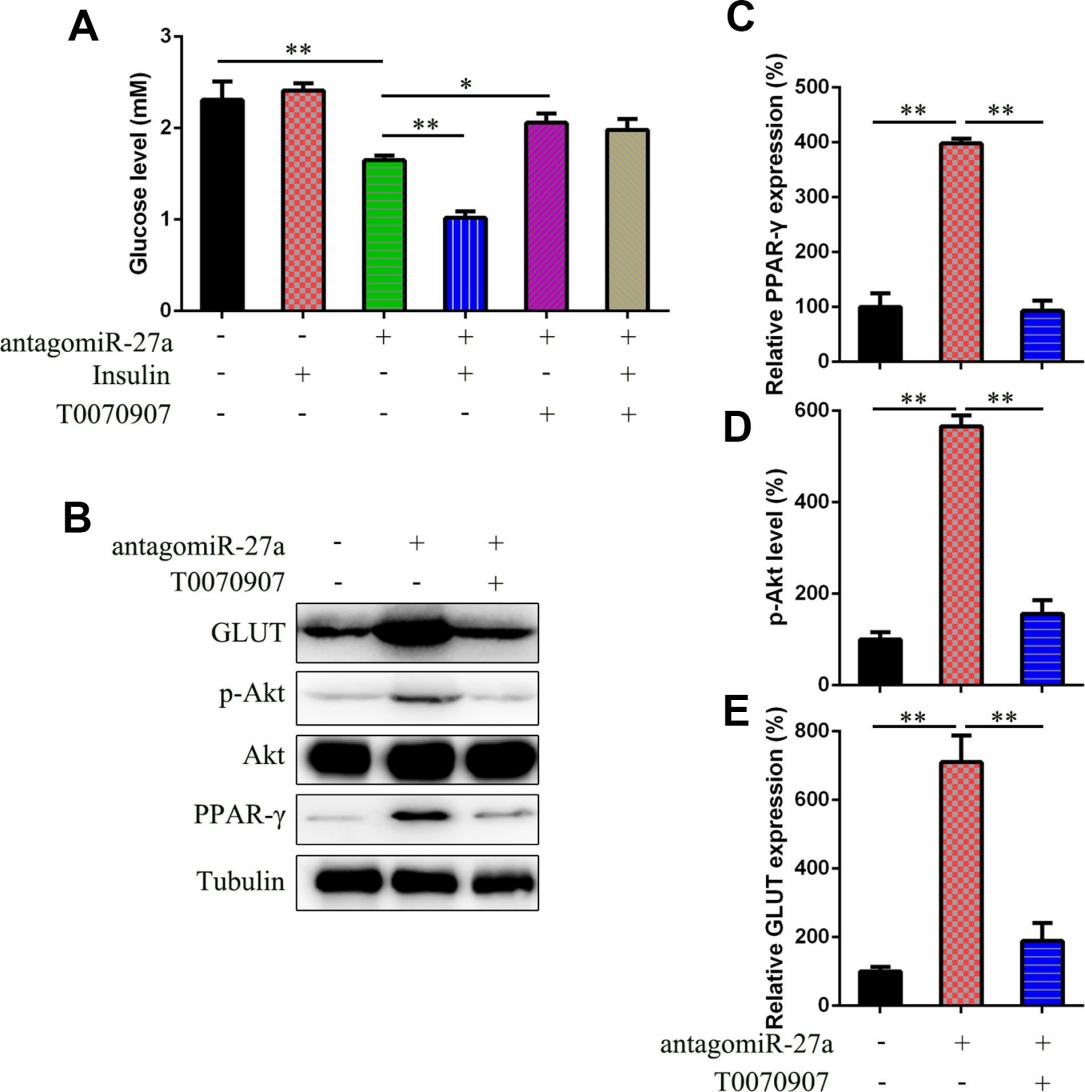
**miR-27a downregulation promote insulin sensitive via targeting PPAR-γ.** (**A**) Glucose level in IR 3T3-L1 cells that underwent different treatment was examined to assess the effects of PPAR-γ inhibitor T0070907 (1 μM) on insulin sensitivity. (**B**) WB was used to detect the PPAR-γ, Akt, GLUT expression, and Akt phosphorylation in cells with or without T0070907 treatment (1 μM) for 24 h. (**C**) qPCR determined that the PPAR-γ expression was inhibited by T0070907. (**D**) Image pixel analysis showed phosphorylated level Akt protein after T0070907 treatment. (**E**) qPCR was performed to confirm that T0070907 treatment downregulated the glucose level, which was induced by agomiR-27a transfection. Number = 3. **P < 0.01, compared to indicated groups.

To confirm the effects of PPAR-γ on miR-27a-regulated insulin sensitivity, antagomiR-27a-transfected IR cells were treated with the PPAR inhibitor T0070907. T0070907 has been used to reduce peroxisome proliferator-activated receptor gamma (PPAR-γ) expression in lipopolysaccharide (LPS)-induced leukemia RAW264.7 cell line [29], therefore it is PPAR-γ specific inhibitor. T0070907 treatment restored the antagomiR-27a-reduced glucose levels (Figure 5A). Furthermore, T0070907 treatment reduced PPAR-γ expression, Akt phosphorylation, and GLUT4 expression induced by antagomiR-27a (Figure 5B–5E).

### MiR-27a regulates insulin sensitivity by activation of PI3K/Akt signaling

**Figure 6:**
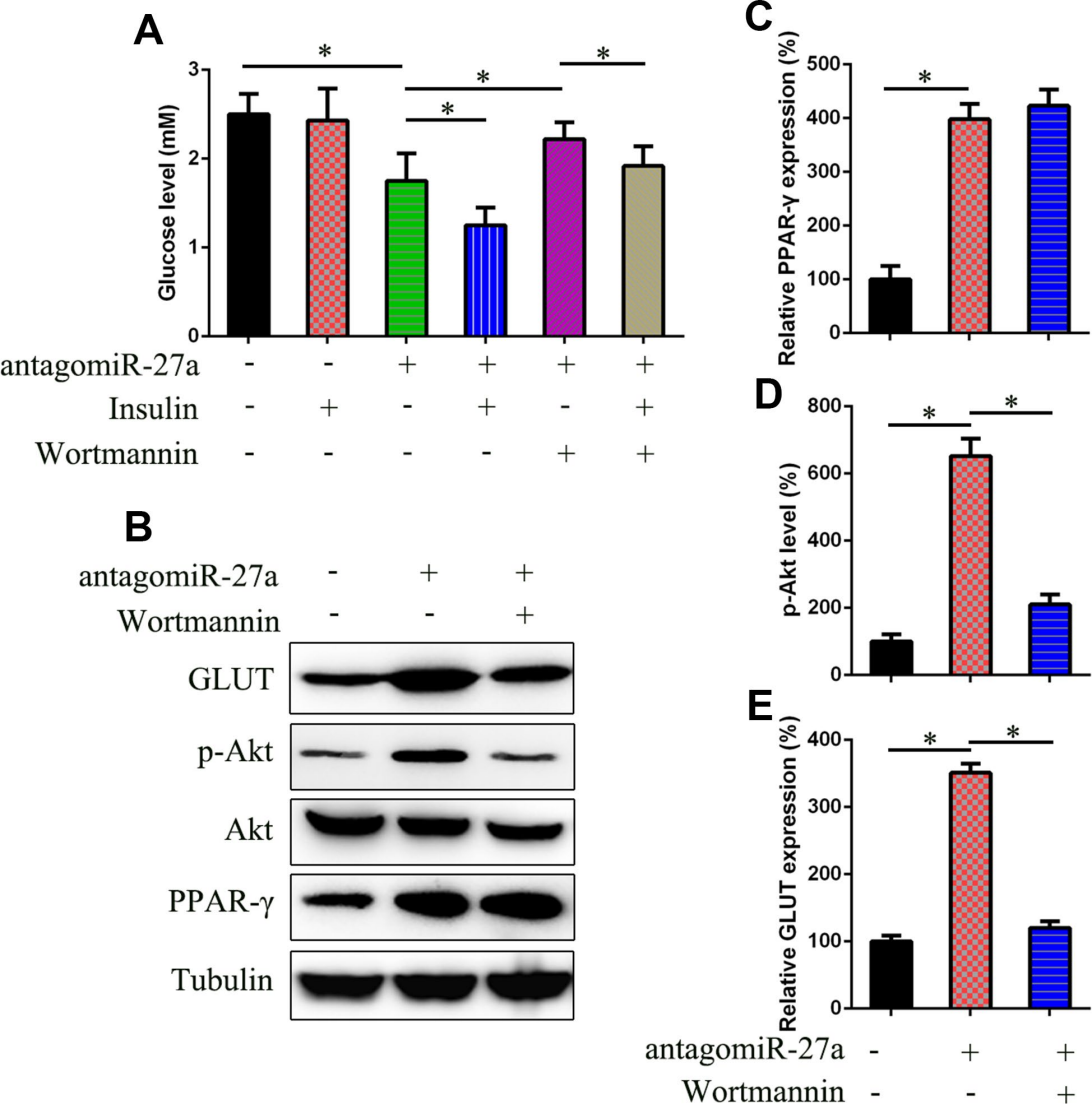
**Effects of miR-27a on the PI3K/Akt signaling pathways in IR 3T3-L1 cells.** (**A**) Glucose level was examined in IR 3T3-L1 cells that underwent different treatments to assess the effects of PI3K inhibitor wortmannin (2.5 μM) on insulin sensitivity. (**B**) WB was performed to examine the PPAR-γ, Akt, GLUT expression, and Akt phosphorylation in cells transfected with agomiR-27a and/or treated with wortmannin. (**C**) qPCR determined that the PPAR-γ expression was not inhibited by wortmannin (2.5 μM) for 24h. (**D**) Image pixel analysis showed phosphorylated level Akt protein after wortmannin treatment. (**E**) qPCR was performed to confirm that wortmannin treatment downregulated the glucose level that was induced by agomiR-27a transfection. Number = 3. *P < 0.05 vs. indicated groups.

AntagomiR-27a-transfected IR cells were treated with the PI3K inhibitor wortmannin to confirm the role of PI3K/Akt signaling in miR-27a-PPAR-γ-regulated insulin sensitivity. The results showed that the reduced level of glucose induced by antagomiR-27a transfection was recovered after wortmannin treatment (Figure 6A). Additionally, there was no change in PPAR-γ expression after treatment, whereas the phosphorylation of Akt and GLUT4 expression were significantly reduced (Figure 6B–6E).

## DISCUSSION

The mechanisms underlying the development of metabolic diseases, such as T2DM, are synergetic processes related to the transcription of various genes T2DM has been investigated in many previous studies; however, the current understanding of the molecular mechanisms behind its development is insufficient. The present study revealed that miR-27a, whose expression is increased in IR adipocytes and HFD-fed obese mice, suppresses the expression of PPAR-γ. Downregulation of miR-27a levels in the cells and the mice enhanced glucose uptake in time- and dose-dependent manners. Further examination demonstrated that miR-27a binds directly to the 3′-UTR of PPAR-γ, thereby repressing its expression. Previous study also indicated that miR-27a binding sites are also present in RXRα [30], both heterodimeric binding partners of PPAR-γ [31]. On the other hand, many other miRs will target PPAR-γ and of course other components of the PI3k pathway [32]. Therefore, we cannot exclude other possibility that miR-27a may mediate IR through other regulation of other target genes. The downregulation of miR-27a augmented GLUT4 expression and PI3K/Akt signaling by controlling PPAR-γ expression. The findings of this study indicate that miR-27a serves as a positive modulator of IR and glucose metabolism by targeting the PPAR-γ gene and suggest it may be a promising target for the treatment of obesity and T2DM.

Several methods can be used to establish an IR model at the cellular level, for instance, dexamethasone administration to FL83B cells, pretreatment of L6 myotubes with high glucose and dexamethasone [33], and treatment of HepG2 cells with palmitate and glucosamine [34]. In the present study, TNF-α was utilized to establish an IR model in 3T3-L1 adipocytes. The presence of a high glucose level, as determined by a glucose detection assay, suggested the successful establishment of the IR cell model. To establish an animal model, we utilized an HFD. An HFD elevates free fatty acid levels, which induce IR in skeletal muscle by the formation of two intermediate metabolites: diacylglycerols (DAGs) and ceramides. DAGs activate members of the protein kinase C family, leading to the activation of serine/threonine kinases that further impair IRS tyrosine phosphorylation. Ceramides in turn mediate the activation of protein phosphatase A2, which can phosphorylate Akt2. Ceramides can also activate inflammatory pathways, including JNK and NFkb, and thus induce IR [35].

MiRNAs modulate the expression of downstream genes in various malignancies and tissues. Many target genes can stimulate mRNA cleavage or reversely modulate translation by binding to miRNAs with their 3′-UTRs. Consequently, our research is partly aimed at the recognition of downstream target genes that are modulated directly by miR-27a. Based on bioinformatics analysis and DLRA, PPAR-γ was confirmed as a target of miR-27a. Our study suggests that miR-27a led to the development of IR in adipocytes and obese mice, at least partially, by targeting the 3′-UTR of PPAR-γ. PPAR-γ has been reported as a transcription factor, because of its nuclear receptor protein property [36]. Additionally, this protein also has key roles in regulating cell differentiation and development, carbohydrate, lipid, and protein metabolism [37], as well as tumorigenesis [38] in higher organisms [39]. Our study revealed that the enhanced expression of PPAR-γ by miR-27a silencing reinforces insulin sensitivity. In addition, the administration of T0070907, a PPAR-γ inhibitor, inhibited the effects of antagomiR-27a transfection, which contributed to the reduction of glucose and the increase of insulin sensitivity in adipocytes. These results indicate that PPAR-γ serves as a target gene to enhance glucose catabolism during T2DM. This observation was also consistent with previous investigations [40, 41].

Akt, a downstream target of PI3Ks (heterodimeric proteins consisting of 85-kDa regulatory and 110-kDa catalytic subunits) [42], is well known for its role in transducing the anti-apoptotic signal by phosphorylating target proteins involved in the regulation of cell proliferation, e.g., ASK1, Bim, Bad, and XIAP, as well as the Foxo3a transcription factor [43–45]. Lipid and protein phosphatases have negative regulating effects on the PI3K pathway. In the insulin-induced signal transduction pathway for glucose metabolism, Akt is phosphorylated after the insulin receptor b subunit is activated [46]. The transfer of glucose into cells is facilitated by the activation of the Akt sensor, where GLUT4 is translocated from the cytoplasm to the plasma membrane [47]. In IR 3T3-L1 adipocytes, glucose uptake was increased due to miR-27a downregulation, which was inhibited when the cells were pre-incubated with the specific PI3K inhibitor wortmannin. These results confirmed that miR-27a exerts its role through the PI3K/Akt pathway.

High serum insulin levels have been reported to be associated with an increased risk of prostate cancer. Previous study has demonstrated the relationship between insulin resistance and prostate cancer risk in Chinese men. Men in the highest tertile of insulin resistance had an increased risk of prostate cancer (odds ratio = 2.78, 95% idence interval = 1.63 to 4.72) [48]. MiR-27a has also been proposed as a target in prostate cancer [49], although other reports showed that miR-27A acted as a tumor suppressor by targeting MAP2K4 and mediated prostate cancer progression [50], suggesting that miR-27a study may be an opportunity to combine therapies for prostate cancer and obesity/other metabolic diseases.

In conclusion, we reported the expression levels of miR-27a in adipocytes treated with TNF-α and in HFD-induced obese mice. MiR-27a plays a considerable role in promoting IR in both of these models, at least partially through the PPAR-γ-mediated PI3K/Akt signaling pathway. Out data suggest that miR-27a itself might be a promising target to improve IR and glucose metabolism during the progression of T2DM.

## MATERIALS AND METHODS

### Animals and treatment

Twenty-four C57BL/6 male mice (Average weight, 20.3 g) were purchased from Vital River (Beijing, China). An HFD-induced obese model was established as previously reported [51]. Briefly, C57BL/6 mice (n = 6, 3-4 weeks old) were fed an HFD (Research Diet; 45% kcal from fat) or standard chow diet (n = 6), for 10 weeks. The experiments were performed under controlled humidity (45–55%) and temperature (20–24 °C).

Mice were injected intravenously via the tail vein with adenovirus encoding green fluorescent protein in the negative control group (AD-NC, n = 6) or with adenovirus miR-27a-3p inhibitor (Ad-miR-27a, n = 6); the dose was 1.0 × 10^8^ plaque-forming units in 0.2 mL phosphate-buffered saline (PBS; 0.2 mL/25 g body weight). The mice were sacrificed on day 9 after adenovirus injection, and the pancreas, liver, and white adipose tissue were harvested for quantitative real-time PCR (qRT-PCR) and western blot (WB) analyses. All animal experiments were approved by the Animal Ethics Committee at The First Affiliated Hospital of Quanzhou, Fujian Medical University and were conducted in compliance with the recommendations in the National Research Council Guide for the Care and Use of Laboratory Animals.

### Cell cultivation and transfection

The mouse 3T3-L1 cell line was provided by the Shanghai Institutes for Biological Sciences, Chinese Academy of Sciences. First, RPMI-1640 medium containing streptomycin (100 mg/mL; Gibco), penicillin (100 U/mL), glutamine (2 mmol/L), and fetal bovine serum (FBS) (10%; Gibco) was used for the cultivation of 3T3-L1 preadipocytes at 37 °C in 5% CO_2_. Differentiation into adipocytes was induced with reference to previous studies [52, 53]. In brief, a differentiation mixture supplemented with dexamethasone (1 μM), insulin (10 μg/mL), and 3-isobutyl-1-methylxanthine (0.5 mM) in Dulbecco’s modified Eagle’s medium (DMEM) with 10% FBS was prepared and used for the stimulation of the 3T3-L1 preadipocytes (after confluence reached 100%) for 48 h. This was followed by 10 days of cell cultivation in DMEM supplemented with insulin (10 μg/mL) and FBS (10%). Finally, light microscopy, along with Oil Red O staining, was performed to confirm the presence of mature adipocytes, which were then used in subsequent experiments.

### Establishment of the IR adipocyte model

IR is an important contributor to the pathogenesis of obesity and T2DM, which is in part induced by TNF-α [54–56]. First, the differentiated 3T3-L1 adipocytes were pretreated with high-glucose DMEM containing FBS (0.5%, w/v) for 3 h. The obtained cells were further treated with high-glucose DMEM containing FBS (10%, w/v) supplemented with TNF-α (10 ng/mL) for 1 day. Subsequently, they were incubated for 0.5 h in high-glucose DMEM containing FBS (10%, w/v) and insulin (100 nM). For comparison, control groups were also established wherein adipocytes were treated before adding insulin and/or TNF-α. To verify the successful construction of the IR cell model, a glucose assay kit (Sigma-Aldrich) was used to detect glucose.

### Recombinant adenovirus preparation

Recombinant adenovirus expressing a miR-27a inhibitor (AD-miR-27a) and a negative control adenovirus vector (AD-NC) were purchased from Shanghai GeneChem Co., Ltd. and used as reported in the literature [57].

### MiR-27a agomiR/antagomiR preparation

AgomiR-27a (, UUC ACA GUG GCU AAG UUC CGC; agomiR-NC, UUC UCC GAA CGU GUC ACG U), antagomiR-27a (, GCG GAA CTT AGC CAC TGT GAA; antagomiR-NC, CAG UAC UUU UUG UGU AGU ACA A), and their scramble controls were acquired from RiboBio. AgomiR/antagomiR-27a mimic/inhibitor and NC mimic/inhibitor were supplemented with 0.9% NaCl to a final concentration of 10 mM for further use.

### Glucose- and insulin-tolerance tests

The mice were fasted for 12 h and injected intraperitoneally with D-glucose (2 g/kg). For the ITT, recombinant human insulin (100 U/mL) was diluted in saline to a concentration of 0.075 U/mL before intraperitoneal injection (0.1 mL/10 g body weight). Blood glucose levels were determined in samples taken from the tail vein at 15, 30, 45, 60, and 90 min using a glucometer (One Touch Ultra).

### Glucose and insulin detection

After washing with PBS five times, 3T3-L1 cells were stimulated for 2 days in DMEM supplemented with insulin (10 μg/mL) and FBS (10%). A glucose assay kit (Sigma-Aldrich) and an insulin enzyme-linked immunosorbent assay kit (Biocompare.com) were employed to detect glucose and insulin concentrations in the medium.

### 2-Deoxyglucose (2-DG) uptake measurement

Glucose uptake tests were performed using a modified protocol, as previously described [52]. Briefly, after treatment, the cells were washed three times with Krebs-Ringer phosphate buffer. The cells were incubated with a final concentration of 1 μCi/mL ^3^H-2-Deoxyglucose (2-DG; GE Healthcare) for 10 min, and the reaction was terminated by washing with Krebs-Ringer phosphate buffer. After the cells were lysed with 0.1 N NaOH, radioactivity in disintegrations per minute (dpm) was determined using a scintillation counter (LS 6500; Beckman). Finally, dpm counts were corrected for the measured protein content using a BCA protein assay in each well.

### Dual-luciferase reporter assay

The 3′-untranslated region (UTR) of the PPAR-γ gene was PCR amplified prior to fusion with the GV126 luciferase gene. The binding site of the PPAR-γ gene as well as miR-27a was ablated via site-directed mutagenesis, which served as a control. The thymidine kinase promoter (pRL-TK vector; TaKaRa) and plasmids expressing Renilla luciferase were used to adjust for transfection efficiency. HEK 293T cells were co-transfected with agomiR-27a and NC using luciferase reporter vectors, and the luciferase assay was conducted.

### Site-directed mutagenesis

Site-directed mutagenesis was carried out to mutate the WT 3’-UTR of PPAR-γ gene by utilizing Fast Mutagenesis System following the instrument of product manual (FM111-01, Transgen Biotech).

### Bioinformatics analysis

Bioinformatics tools, namely, miRDB (http://mirdb.org/) and TargetScan (http://www.targetscan.org), were utilized to search for the putative target of miR-27a.

### Western blot analysis

Cell lysis was performed with a protease suppressor cocktail (Roche) in a pH 8.0 RIPA buffer containing sodium dodecyl sulfate (SDS; 0.1%), NP-40 (1%), NaCl (150 mM), and Tris-HCl (50 mM). A BCA Protein Quantitation Kit was employed for the quantification of proteins. The protein samples were separated using SDS-polyacrylamide gel electrophoresis (10%) and transferred to polyvinylidene fluoride membranes (0.45 μm). The membranes were blocked for 60 min in PBS-Tween-20 (PBST) containing 5% bovine serum albumin (BSA) at room temperature. The membranes were incubated with anti-actin, anti-Bax, anti-Bcl-2, and anti-CDC14B antibodies at 4 ºC for 60 min. The membranes were further incubated with secondary goat anti-rabbit immunoglobulin G (1:10,000) or goat anti-mouse immunoglobulin G (1:10,000) antibodies conjugated to Amersham ECL peroxidase at room temperature for 60 min. Immunoreactivity was measured on a C-DiGit Blot Scanner with a Super Signal West Femto Maximum Sensitivity Substrate Kit (Thermo).

### RNA extraction and qRT-PCR

Total RNA was isolated from the cells using TRIzol reagent. Transcription levels were analyzed with the Roche Light-Cycler 480 Real-Time PCR system. The internal reference was glyceraldehyde 3-phosphate dehydrogenase (GAPDH). A SYBR Green PCR Master Mix was employed for qRT-PCR (20 μL). The following cycling conditions were used for PCR: initial denaturation for 10 min at 95 °C, 40 cycles of denaturation for 15 s at 95 °C, annealing for 30 s at 60 °C, and extension for 30 s at 72 °C. Quantification was performed according to the 2^-ΔΔCT^ method with normalization to GAPDH, which was measured relative to a calibrator (mean of the control samples).

### Immunofluorescence analysis

Virus infection of IR cells grown in 24-well plates with cover slides was performed at a multiplicity of infection of 0.1 TCID_50_/cell. Paraformaldehyde (4%) in PBS was used to fix the cells for 1 h at room temperature. The cells were permeabilized with PBST for 10 min at 25 °C, followed by a 1-h incubation in PBST (including 0.4% BSA) at 37 °C and a subsequent 1-h incubation with polyclonal anti-VP1 antibody, which was diluted in PBST (including 0.2% BSA) at 37 °C. Following a 1-h wash with PBST, the cells were incubated for 1 h with TRITC-labeled goat anti-rabbit antibody diluted in 0.2% BSA and PBST at 37 °C. The cells were washed for 1 h with PBST. Cell nuclei were stained using DAPI. A confocal laser scanning fluorescence microscope (LSCMFV500; Olympus) was employed to analyze VP1 staining in the cells.

### Statistical analysis

The results are expressed as the mean ± standard deviation. One-way analysis of variance or a two-tailed Student’s t-test was employed for intergroup comparisons. P < 0.05 was considered to indicate statistical significance.
